# 0.5 V Fifth-Order Butterworth Low-Pass Filter Using Multiple-Input OTA for ECG Applications

**DOI:** 10.3390/s20247343

**Published:** 2020-12-21

**Authors:** Montree Kumngern, Nattharinee Aupithak, Fabian Khateb, Tomasz Kulej

**Affiliations:** 1 Department of Telecommunications Engineering, Faculty of Engineering, King Mongkut’s Institute of Technology Ladkrabang, Bangkok 10520, Thailand; montree.ku@kmitl.ac.th (M.K.); yoknattharinee@gmail.com (N.A.); 2 Department of Microelectronics, Brno University of Technology, Technicka 3058/10, 612 00 Brno, Czech Republic; 3 Faculty of Biomedical Engineering, Czech Technical University in Prague, nám. Sítná 3105, 272 01 Kladno, Czech Republic; 4 Department of Electrical Engineering, Czestochowa University of Technology, 42-201 Czestochowa, Poland; kulej@el.pcz.czest.pl

**Keywords:** fifth-order low-pass filter, operational transconductance amplifier, multiple-input bulk-driven technique, subthreshold region, nanopower

## Abstract

This paper presents a 0.5 V fifth-order Butterworth low-pass filter based on multiple-input operational transconductance amplifiers (OTA). The filter is designed for electrocardiogram (ECG) acquisition systems and operates in the subthreshold region with nano-watt power consumption. The used multiple-input technique simplifies the overall structure of the OTA and reduces the number of active elements needed to realize the filter. The filter was designed and simulated in the Cadence environment using a 0.18 µm Complementary Metal Oxide Semiconductor (CMOS) process from Taiwan Semiconductor Manufacturing Company (TSMC). Simulation results show that the filter has a bandwidth of 250 Hz, a power consumption of 34.65 nW, a dynamic range of 63.24 dB, attaining a figure-of-merit of 0.0191 pJ. The corner (process, voltage, temperature: PVT) and Monte Carlo (MC) analyses are included to prove the robustness of the filter.

## 1. Introduction

**Figure 1:**
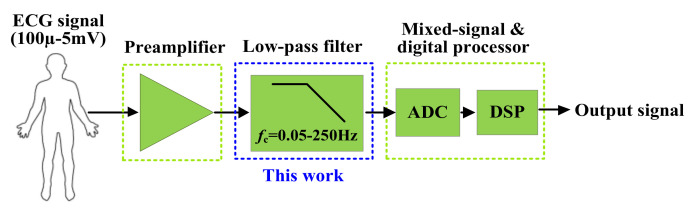
Electrocardiogram acquisition system.

Continuous-time filters are widely used in biomedical systems devoted to applications in electroencephalographic (EEG), electromyographic (EMG), and electrocardiographic (ECG) systems. The biological signals processed in these systems typically occupy the frequency range of 0.05–250 Hz, with an amplitude of 15 µV–5 mV [1]. In more detail, the frequency/amplitude ranges for EEG, EMG, and ECG signals are 0.05–60 Hz/15−100 µV, 10−200 Hz/0.1−5 mV, and 0.05−250 Hz/100 µV−5 mV, respectively. Figure 1 shows a typical data acquisition system for ECG signal processing. The pre-amplifier stage amplifies a low-amplitude ECG signal, then the low-pass filter selects the frequency range and eliminates out-of-band noise. The filtered analog signal is converted into digital form by an analog-to-digital converter (ADC) and then it is further processed by a digital signal processing (DSP) block. This work focused on the design of a low-pass filter with the cutoff frequency of 250 Hz. The analog low-pass filters for ECG acquisition systems should be designed to meet specific requirements, such as high dynamic range, low-power consumption, and small chip area. There are many low-pass filters for ECG acquisition systems described in the literature [2,3,4,5,6,7,8,9,10]. The Butterworth approximation is usually used because it provides a better linear phase and flat response within each bandwidth. Considering the analog filters in [2,3,4,5,6,7,8,9,10], one can distinguish two main techniques that have been used to realize the low-pass Butterworth filters: the cascade approach [2,3,4,5,6] and the ladder simulation approach [7,8,9,10]. The cascade structure can be obtained by cascading several biquad filters, which leads to a simple and easy-to-tune realization.

**Figure 2:**
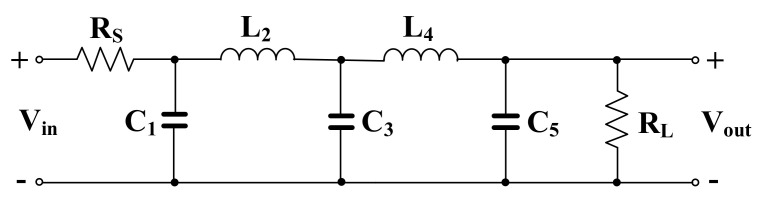
Prototype of a fifth-order low-pass filter.

The present work focused on the second approach, i.e., the ladder simulation of a prototype filter. In particular, we designed a fifth-order low-pass Butterworth filter based on the RLC prototype shown in Figure 2. As it is widely known, the high-order filters based on the RLC prototypes have lower pass-band sensitivity to the variation of passive elements, compared with that of the cascade approach.

**Figure 3:**
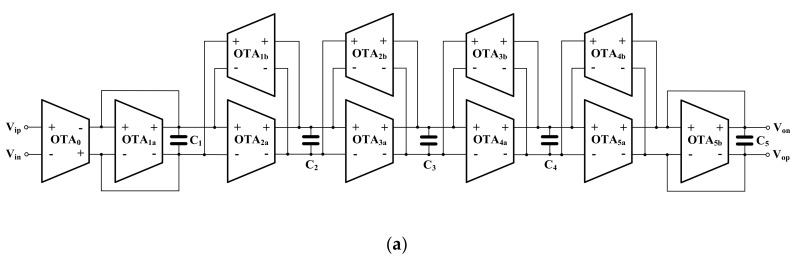

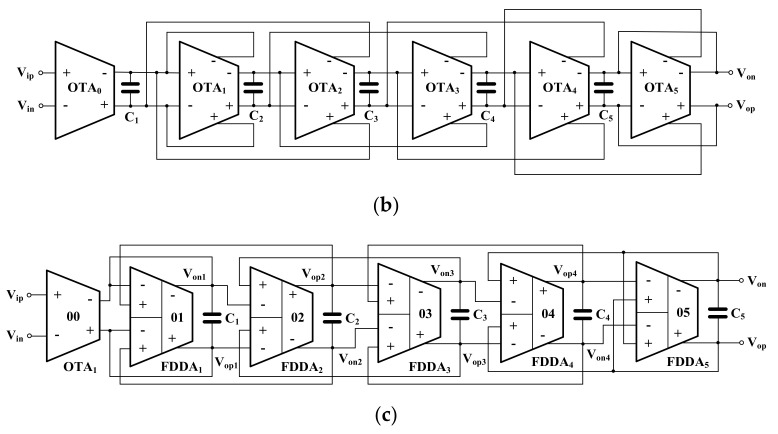
Fifth-order Butterworth low-pass filters, (**a**) FD-OTA-C filter [7], (**b**) MOFD-OTA-C filter [8,9], (**c**) FDDA-based filter [10].

The fifth-order low-pass Butterworth filters derived from the LC ladder-type filter were reported in [7,8,9,10]. The fifth-order Butterworth low-pass filter using fully differential operational transconductance amplifiers (FD-OTAs) is shown in Figure 3a [7]. The floating inductors L_2_ and L_4_ are simulated using OTA-based gyrators. The resistors R_S_ and R_L_ are simulated using OTAs as well. It should be noted that the filter in [7] employs eleven FD-OTAs and consumes 453 nW of power. The number of active devices that are used to realize this fifth-order Butterworth filter can be reduced by using multiple-output fully differential OTA (MOFD-OTA) as shown in Figure 3b [8,9], or fully differential-difference transconductance (FDDA) (a multiple-input active device) as shown in Figure 3c [10]. The structures in [8,9] employ six MOFD-OTA while the structure in [10] employs five FDDAs and one OTA. The filter in [8] consumes 350 nW of power and offers a 49.9 dB dynamic range while the filter in [9] consumes 41 nW of power and offers a 61.2 dB dynamic range. The filter in [10] consumes 453 nW of power and offers a 50 dB dynamic range.

This paper proposes a fifth-order Butterworth low-pass filter based on multiple-input operational transconductance amplifiers. It is clearly shown that the number of active devices needed to realize the fifth-order low-pass filter can be reduced by using the multiple-input OTA and results in reducing the power consumption and the active chip area. A novel technique with a multiple-input gate-driven (MIGD) transistor is used to realize multiple-input OTA with an internal CMOS structure as simple as a conventional OTA, hence, no additional current branches or cascade connections of multiple OTAs is needed. Unlike the floating-gate technique, the multiple-input technique does not require any additional processing steps to eliminate the trapped charge effect on the isolated gate nor any auxiliary circuit. Another advantage is that the multiple-input gate-driven P- or N-MOS transistors can be realized with any CMOS process. It is worth noting that the results presented in this work are based on pre-layout simulation and this work does not include the physical realization of the filter, nor the experimental testing in the context of ECG applications. However, the principle of multiple-input transistors, as multiple-input bulk-driven and multiple-input bulk-driven quasi-floating-gates, have been confirmed experimentally by Khateb et al. in previous works [11,12,13]. The paper is organized as follows: Section 2 shows the principle of multiple-input gate-driven OTA and the filter design based on it, Section 3 the simulation results, and finally Section 4 the conclusion.

## 2. Fifth-Order Butterworth Low Pass Filter

### 2.1. Multiple-Input Gate-Driven OTA

**Figure 4:**
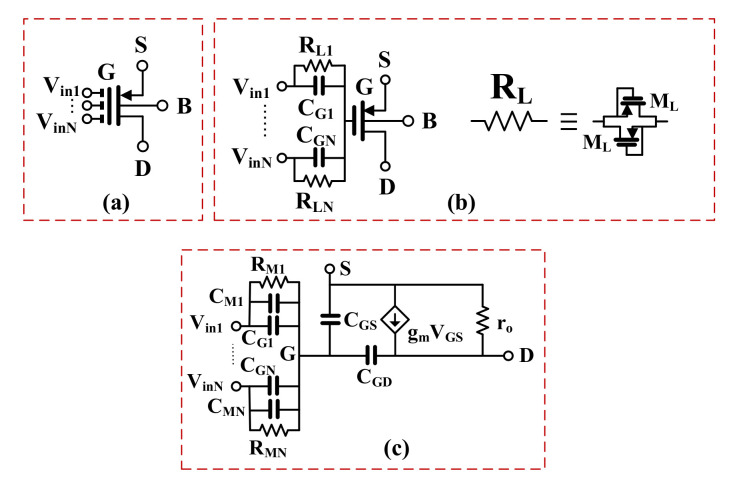
MIGD MOS transistor, (**a**) symbol, (**b**) realization, (**c**) small-signal model.

The active filter proposed in this work exploits multiple-input OTAs, which allows for simplifying its overall structure [14]. The multiple-input OTA is realized using a concept of a multiple-input MOS transistor. The symbol and CMOS realization of this element are shown in Figure 4a,b, respectively. As it is seen in Figure 4b, the multiple-input MOS can be seen as a connection of an “internal” MOS transistor and a voltage divider/analog summing circuit, composed of capacitances $C_{Gi}$ (i = 1…N). The capacitors $C_{Gi}$ are shunted by the large resistances $R_{Li}$, which ensures proper biasing of the gate terminal of the internal MOS for DC. The large resistances can be realized using an anti-parallel connection of two minimum-size MOS transistors operating in a cutoff region, as shown in Figure 4b. The small-signal equivalent circuit of the resulting multiple-input MOS is shown in Figure 4c. Assuming $1/\omega C_{Gi}\ll R_{Li}$, the gate potential $V_{G}$ is given by

(1) $$V_{G}=\overset{N}{\underset{i=1}{\sum }}\frac{C_{Gi}}{C_{\sum }}V_{ini}$$

where $C_{\Sigma }$ is the sum of the capacitances $C_{Gi}$ and the input capacitance of an internal MOS seen from its gate terminal $C_{in}$:

(2) $$C_{\sum }=C_{in}+\overset{N}{\underset{i=1}{\sum }}C_{Gi}$$

Since the AC signal at the gate of the internal MOS transistor is attenuated by the capacitive divider, the transconductance of the multiple-input device seen from its i-th input, and operating in the subthreshold region, can be expressed as:

(3) $$g_{mi}=\frac{I_{D}}{n_{p}U_{T}}\cdot \frac{C_{Gi}}{C_{\sum }}$$

where $I_{D}$ is the DC drain current, $n_{p}$. is the subthreshold slope, and $U_{T}$. is the thermal potential. As it is seen from (3), the transconductance seen from the i-th input is equal to the transconductance of the internal MOS, multiplied by the voltage gain of the capacitive voltage divider.

The lower input transconductance $g_{mi}$ entails a lower intrinsic voltage gain of the multiple-input MOS, as well as an increased input-referred noise. Both parameters are degraded by the factor of $C_{\Sigma }/C_{Gi}$. However, it is worth noting that the linear range for such a device is also increased by the factor of $C_{\Sigma }/C_{Gi}$, therefore, its dynamic range (DR) remains the same as that of the internal MOS.

**Figure 5:**
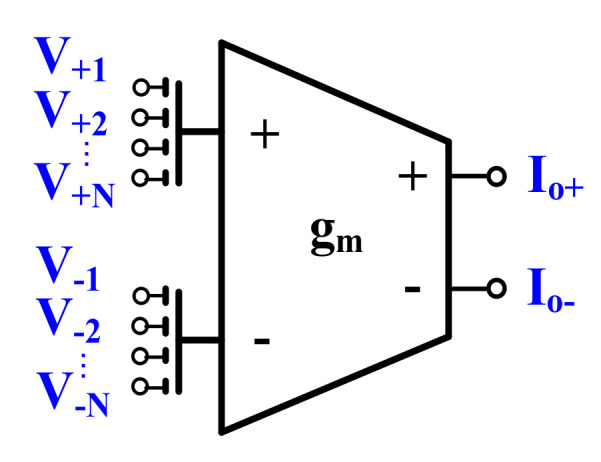
Symbol of a multiple-input operational transconductance amplifier (OTA).

**Figure 6:**
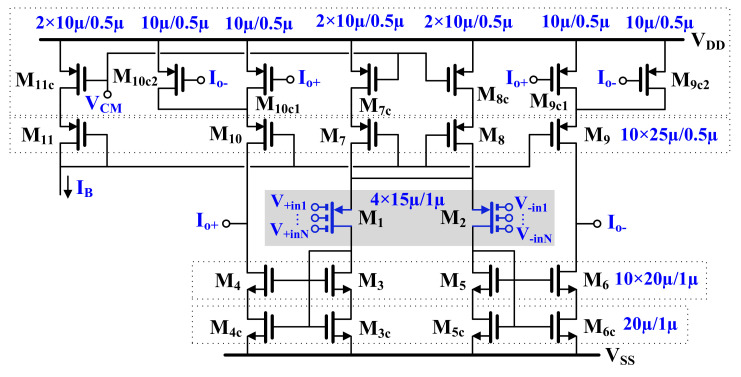
CMOS implementation for an MIGD OTA.

**Figure 7:**
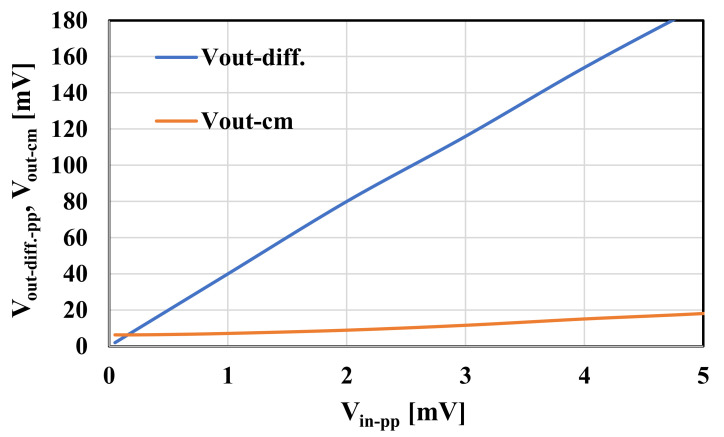
Output differential voltage and common-mode level versus input differential voltage for unloaded OTA in Figure 6.

The multiple-input MOS transistors were used to design a multiple-input OTA. The symbol and CMOS realization of the circuit are shown in Figure 5 and Figure 6, respectively. The multiple-input MOS transistors M_1_ and M_2_ were used to create a multiple-input differential pair, biased by the self-cascode current sources M_7,7c_ and M_8,8c_. The drain currents of the input differential pair are transferred to the outputs (I_o+_ and I_o-_) through the current mirrors composed of the self-cascode transistors M_3/3c_-M_4/4c_ and M_5,5c_-M_6,6c_. The current mirrors are loaded with the self-cascode current sources M_10,10c_ and M_9,9c_. Note that the tail node that supplies the differential pair in Figure 6 is drawn with two branches for esthetic reasons. The application of self-cascode connections in this design allows for an increase in the output resistance of the OTA, which entails increasing the DC voltage gain of this circuit. The transistors M_9c_-M_11c_ form a simple common-mode feedback circuit (CMFB) circuit, which forces the output common-mode level to be equal to the reference potential V_CM_. All the transistors operate in a subthreshold triode region. If the common-mode level is increasing/decreasing, the channel resistances of M_10C1,c2_ are increasing/decreasing as well, thus lowering the currents flowing through M_10_ and M_9_, and consequently, decreasing/increasing the common-mode level to the desired value. The transistors M_9c_ and M_10c_ are divided into two devices, which makes the circuit insensitive to the output differential signals of the OTA, at least for small amplitudes of the signal. For larger amplitudes of the output signals, one can observe nonlinear components of the drain currents I_D9_ and I_D10_, caused by the differential output voltage of the OTA. However, this nonlinear effect is not apparent at the differential output of OTA, since variation of I_D9_ and I_D10_ are identical. This effect, however, causes variation of the output common-mode level. Figure 7 illustrates the large signal transfer characteristics and the common-mode level variation for unloaded OTA in Figure 6 controlled with differential signals. Note, moderate nonlinear effects are caused by the nonlinear output conductance of the OTA rather than that of the CMFB. Variations of the common-mode output voltage are maintained at an acceptable level.

One can say that the applied CMFB has a simple structure and does not consume additional power from supply rails. On the other hand, it slightly limits the maximum output voltage swing due to nonzero voltage drops across transistors M_9c_–M_11c_ and variations of the output common-mode level caused by differential signals. However, the negative effects can be maintained at an acceptable level.

Assuming $1/\omega C_{Gi}\ll R_{Li}$, the differential output current of the OTA can be expressed as:

(4) $$I_{o+}-I_{o-}=I_{B}tanh\left(\overset{N}{\underset{i=1}{\sum }}\frac{V_{+ini}-V_{-ini}}{n_{p}U_{T}}\cdot \frac{C_{Gi}}{C_{\sum }}\right)$$

where $I_{B}$ is the biasing current (it was assumed that $I_{D7}$ = $I_{D8}$ = $I_{D11}$). From (4), the small-signal transconductance from i-th input is given by:

(5) $$g_{mi}=\frac{I_{D}}{n_{p}U_{T}}\cdot \frac{C_{Gi}}{C_{\sum }}$$

The DC voltage gain of the OTA from the i-th input can be expressed as:

(6) $$A_{vd}=g_{mi}r_{out}$$

where $r_{out}$ is the output resistance of the OTA, given by:

(7) $$r_{out}≅g_{m4,6}r_{ds4,6}r_{ds4c,6c}||g_{m9,10}r_{ds9,10}(r_{ds9,10c}/2)$$

Thanks to the self cascode connections, the voltage gain of the OTA can be at an acceptable level, despite the lower transconductance of the input differential pair.

From (4), the third order harmonic distortion of the OTA for a sinusoidal signal applied to one pair of input terminals, while the other pairs are shorted to ground the AC signals, can be expressed as:

(8) $$HD_{3}=\frac{1}{48}{\left(\frac{V_{+i}-V_{-i}}{n_{p}U_{T}}\cdot \frac{C_{Gi}}{C_{\sum }}\right)}^{2}$$

Thus, as it is seen from (8), the input linear range is increased by the factor of $C_{\Sigma }/C_{Gi}$, i.e., the voltage attenuation factor introduced by the input capacitive divider.

The input referred noise of the OTA, including both thermal and flicker noise components, can be expressed as:

(9) $$\bar{v_{nt𝚤}^{2}}=2{\left(\frac{U_{T}}{I_{B}}\right)}^{2}{\left(\frac{C_{\sum }}{C_{Gi}}\right)}^{2}\left[\bar{{𝚤}_{1,2}^{2}}+2\bar{{𝚤}_{3-6}^{2}}+\;2\bar{{𝚤}_{9,10}^{2}}\right]$$

where:

(10) $$\bar{{𝚤}_{1,2}^{2}}=2qI_{B}+\frac{K_{Fp}{\left(\frac{I_{B}}{U_{T}}\right)}^{2}}{fC_{OX}{\left(WL\right)}_{1,2}}$$

(11) $$\;\;\;\bar{{𝚤}_{3-6}^{2}}=4kTg_{ds3-6c}\left(1+\frac{2}{3}\frac{g_{ds3-6c}}{g_{m3-6}}\right)\frac{{\left(g_{m3-6}r_{ds3-6c}\right)}^{2}}{{\left(1+g_{m3-6}r_{ds3-6c}\right)}^{2}}\;\;\;\;+\;\frac{1}{4}\cdot \frac{K_{Fn}{\left(\frac{I_{B}}{U_{T}}\right)}^{2}}{fC_{OX}[\left(WL{)}_{3-6eff}\right]}$$

(12) $$\;\;\;\bar{{𝚤}_{10-14}^{2}}=4kTg_{ds9-10c}\left(1+\frac{2}{3}\frac{g_{ds9-10c}}{g_{m9-10}}\right)\frac{{\left(g_{m9-10}r_{ds9-10c}\right)}^{2}}{{\left(1+g_{m9-10}r_{ds9-10c}\right)}^{2}}\;\;\;\;+\;\frac{1}{4}\cdot \frac{K_{Fp}{\left(\frac{I_{B}}{U_{T}}\right)}^{2}}{fC_{OX}[\left(WL{)}_{9,10eff}\right]}$$

where $g_{ds9-10c}\;\;=\;g_{ds9-10c1}//g_{ds9-10c1}$, WL_ieff_ = (WL_i_∙WL_ic_)/(WL_i_ + WL_ic_), i = 3…10, WL_9,10c_ = WL_9,10c1_ + WL_9-10c2_, K_Fn_ and K_Fp_ are the flicker noise constants for n- and p-channel transistors, respectively, and C_OX_ is the oxide capacitance per unit area.

As it is easy to note from (9), the input referred noise is increased by the factor of $C_{\Sigma }/C_{Gi}$, as compared with the input noise of a single-input OTA biased with the same current. However, if the multiple input OTA is realized with N identical OTAs, each biased with the current of $I_{B}/N$, then the input transconductance from each input and the input referred noise would be the same as that for the proposed realization (see the Appendix A). Since the linear range in the proposed design is increased $C_{\Sigma }/C_{Gi}$ times, then the DR of the proposed solution is also increased in the same proportion. The improved DR can be considered as the most important advantage of the proposed approach. Note that a similar capacitive attenuation approach that increase the dynamic range of OTAs has been presented before [15].

### 2.2. Proposed Filter

**Figure 8:**
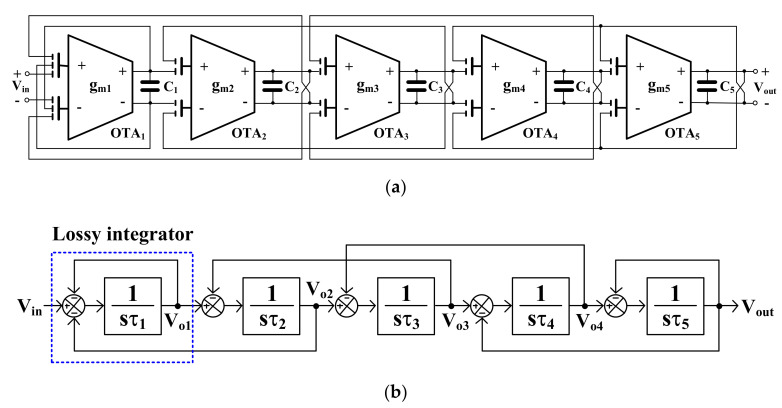
(**a**) Proposed fifth-order Butterworth low-pass filter, (**b**) signal flow graph.

The proposed fifth-order Butterworth low-pass filter is shown in Figure 8a. It was developed from the LC-ladder filter based on the OTA-C topology. Its signal flow graph is shown in Figure 8b, where $\tau_{1}$ = $C_{1}/g_{m1}$, $\tau_{2}$ = $C_{2}/g_{m2}$, $\tau_{3}$ = $C_{3}/g_{m3}$, $\tau_{4}$ = $C_{4}/g_{m4},$ and $\tau_{5}$ = $C_{5}/g_{m5}$. The filter comprises five MIGD OTAs and five capacitors. The number of active devices is reduced from 6 to 5, as compared with [8,9,10], which allows for the reduction of the active area and power.

**Figure 9:**
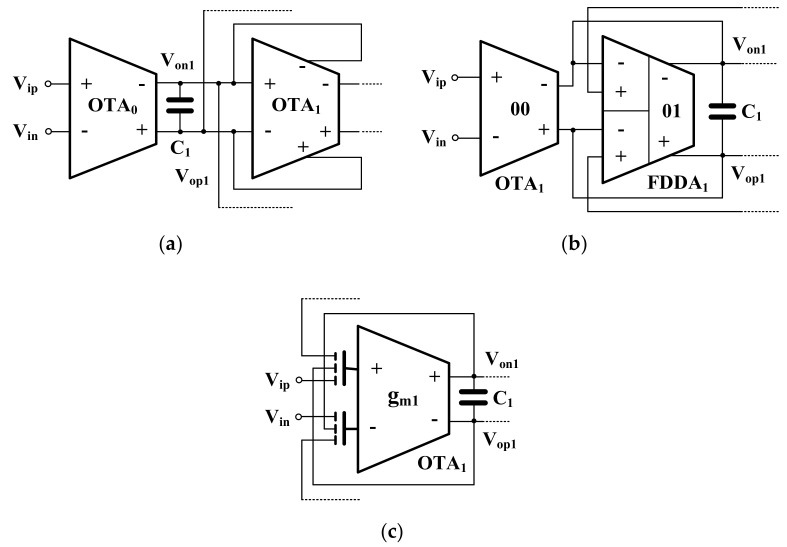
Lossy integrator, (**a**) circuit in [8], (**b**) circuit in [10], (**c**) proposed circuit.

Considering OTA_0_, OTA_1_ in Figure 3b and OTA_0_, FDDA_1_ in Figure 3c, it can be noted that these devices are used to realize a floating resistor [9]. In this work these components together with the capacitor C_1_ create a lossy integrator as shown in Figure 9a [8], Figure 9b [10]. The ideal transfer function of these circuits can be expressed as:

(13) $$\frac{V_{op1}-V_{on1}}{V_{ip}-V_{in}}=\frac{g_{mo}/g_{m1}}{\left(sC_{1}/g_{m1}\right)+1}$$

It is evident that the circuits work as lossy integrators, where the voltage gain can be controlled by $g_{mo}$. Usually, all transconductances are set to be equal for easy tuning. Figure 9c shows the lossy integrator based on the three-input OTA that is proposed in this paper. The ideal transfer function of the circuit in Figure 9c can be expressed as:

(14) $$\frac{V_{op1}-V_{on1}}{V_{ip}-V_{in}}=\frac{1}{\left(sC_{1}/g_{m1}\right)+1}$$

Thus, the circuit works as a lossy integrator with unity gain. Assuming that $g_{mo}$ = $g_{m1}$, Equations (13) and (14) will be identical. Thus, it can be concluded that the OTA_0_ in Figure 3b,c can be removed by using multiple-input OTA. This application can only be realized using multiple-input OTA and it is not possible by using conventional OTA. It should be noted that only the parts mentioned above in Figure 9a of [8], Figure 9b of [10] are modified, the other parts (OTA_2-5_ or FDDA_2-5_) are not changed and the feedback connection is still similar to the filters in [8,10].

## 3. Results and Discussion

The circuit was designed in the Cadence environment using a TSMC 0.18 µm CMOS process with a metal-insulator-metal (MIM) capacitor. The OTA with bias current I_B_ = 3.3 nA consumes 8.25 nW under a 0.5 V supply voltage. The isolation between OTA inputs is assured by the large value resistance of the MOS transistor operating in a cutoff region. The input currents are well below 100 pA for input range rail-to rail.

**Figure 10:**
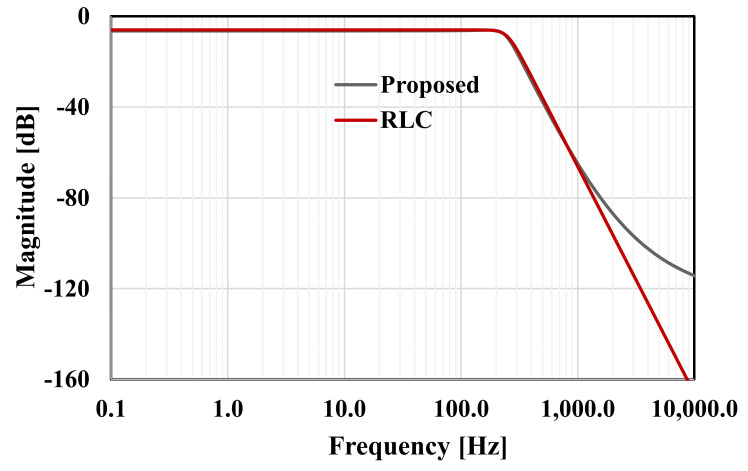
The frequency response of the RLC and the proposed filter.

**Figure 11:**
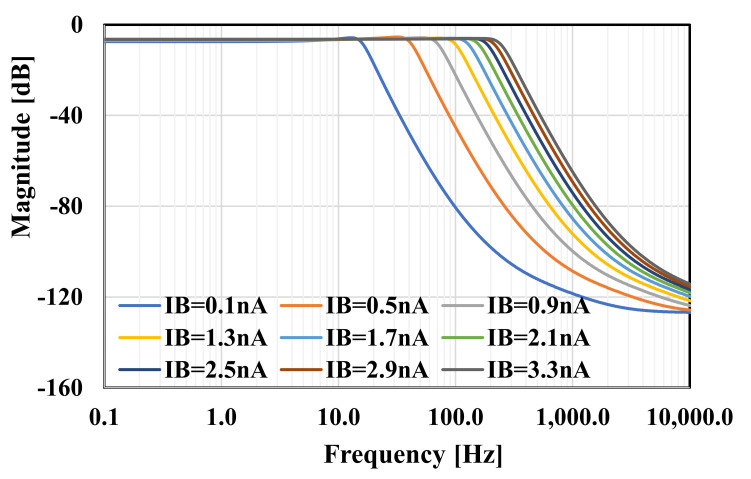
The frequency response of the proposed filter with different bias currents.

**Figure 12:**
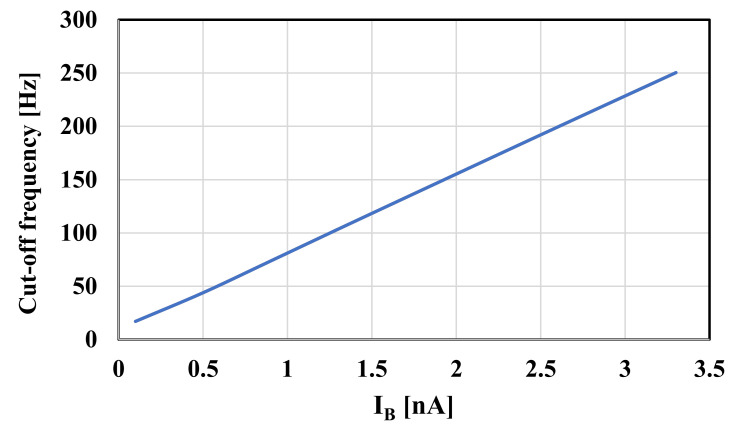
The cut-off frequency versus the bias current.

**Figure 13:**
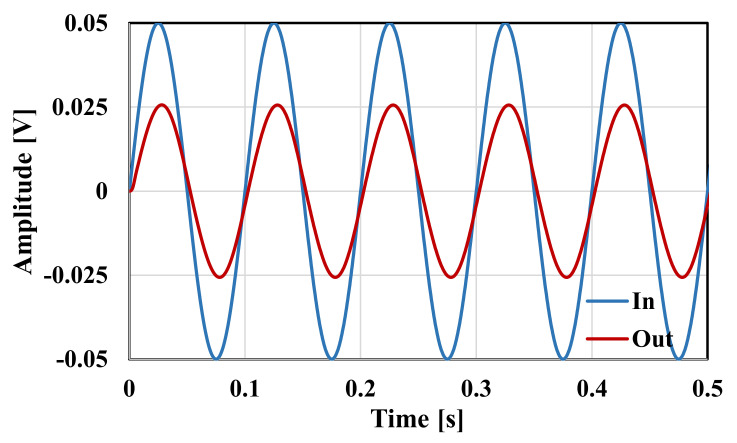
The transient response of the filter for input sine wave with V_inpp_ = 100 mV and 10 Hz.

The RLC filter in Figure 2 was designed for the cut-off frequency of 250 Hz. The prototype element values were chosen as follows: R_S_ = R_L_ = 1 Ω, C_1_ = C_5_ = 393.4 µF, C_3_ = 1.27 mF, and L_2_ = L_4_ = 1.03 mH. For the OTA-C filter C_1_ = C_5_ = 5.43 pF, C_2_ = C_4_ = 14.2 pF, C_3_ = 17.57 pF, and the bias current for each OTA was I_B_ = 3.3 nA. Note that the bias current circuit serves to bias all OTAs hence the maximum power consumption of the filter is 34.65 nW. Figure 10 shows the frequency responses of the RLC and the proposed filter. The gain magnitude at low frequency was −6 dB and −6.4 dB and the cut-off frequency (*f_c_*) was 250.2 Hz and 250.4 for the RLC and OTA filters, respectively. Both curves are in good agreement up to −70 dB. Figure 11 shows the frequency response of the filter with different bias currents ranging from 0.1 nA to 3.3 nA while the *f_c_* was in the range of 17.11 Hz to 250.4 Hz. The tuning capability and the linear relation between *f_c_* and I_B_ are demonstrated in Figure 12. The transient response of the filter for the input sine wave of V_inpp_ = 100 mV and 10-Hz frequency are illustrated in in Figure 13. The total harmonic distortion (THD) was 1%.

**Figure 14:**
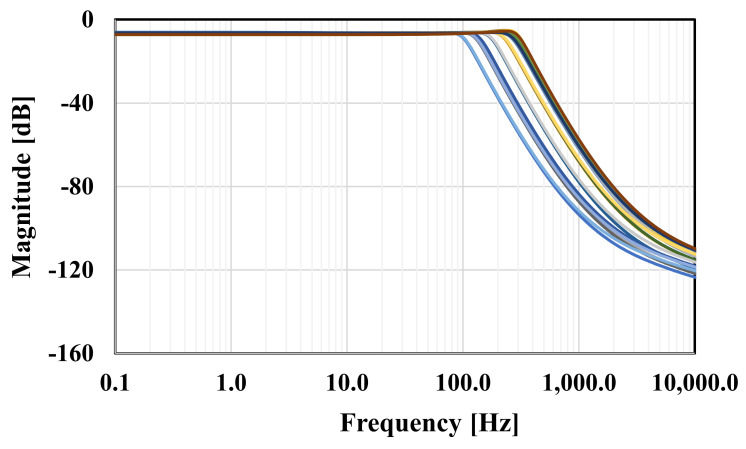
The frequency response of the proposed filter under process, voltage and temperature (PVT) corners.

To check the influence of the process, voltage, and temperature (PVT) variations on the filter performance, the corner analysis was performed. The MOS transistor corners (ss, sf, fs, ff), MIM capacitor corners (ss, ff), voltage supply corners (490 mV, 510 mV), and temperature corners (0 °C, 60 °C) were used. The variation of the gain was in the range of −7.2 dB to −6.13 dB while the variation of the cut-off frequency was in the range of 100.6 Hz to 326.7 Hz, as shown in Figure 14. Note that the temperature corner has the most effect of the variation of the frequency response since the circuit operates in a subthreshold region. However, since the circuit is proposed for biomedical applications it is expected that the temperature variation will be less than the chosen temperature corners. Although the variation of the cut-off frequency is large, the needed value can be simply re-adjusted by the bias current. Note that the amplitudes of the bumps at low bias currents in Figure 11 and at higher frequencies in Figure 14 do not exceed 1.6 dB and do not affect stability of the circuit in a significant manner.

**Figure 15:**
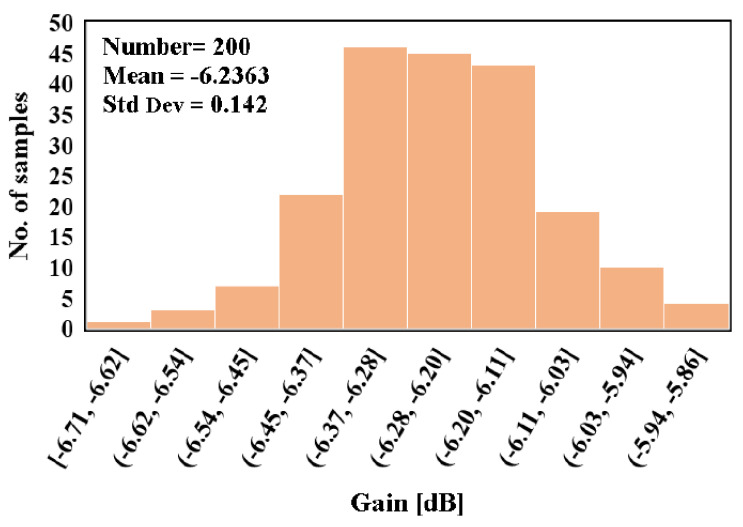
Monte Carlo simulation of the voltage gain.

**Figure 16:**
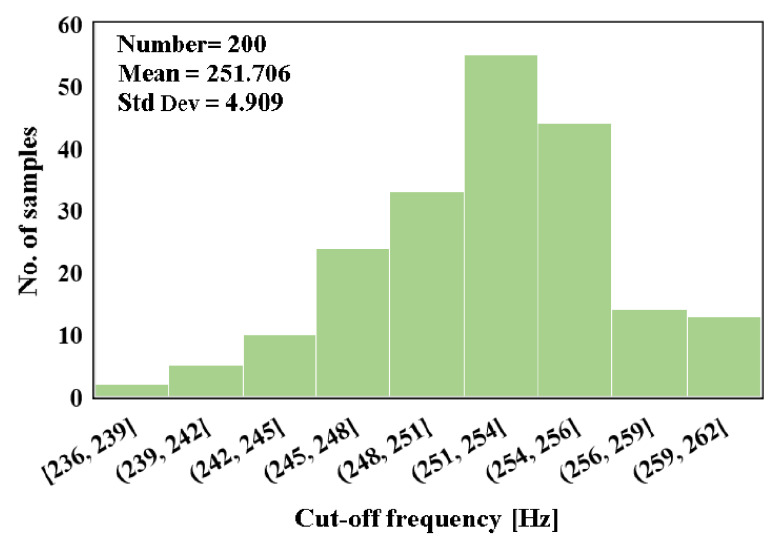
Monte Carlo simulation of the cut-off frequency.

**Figure 17:**
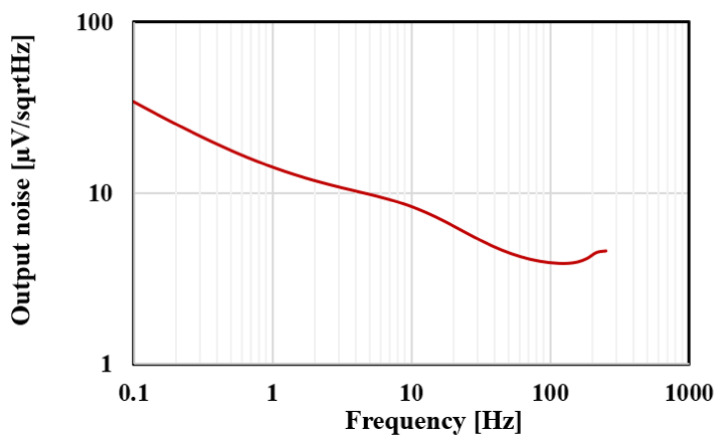
The output referred noise density of the proposed filter.

**Figure 18:**
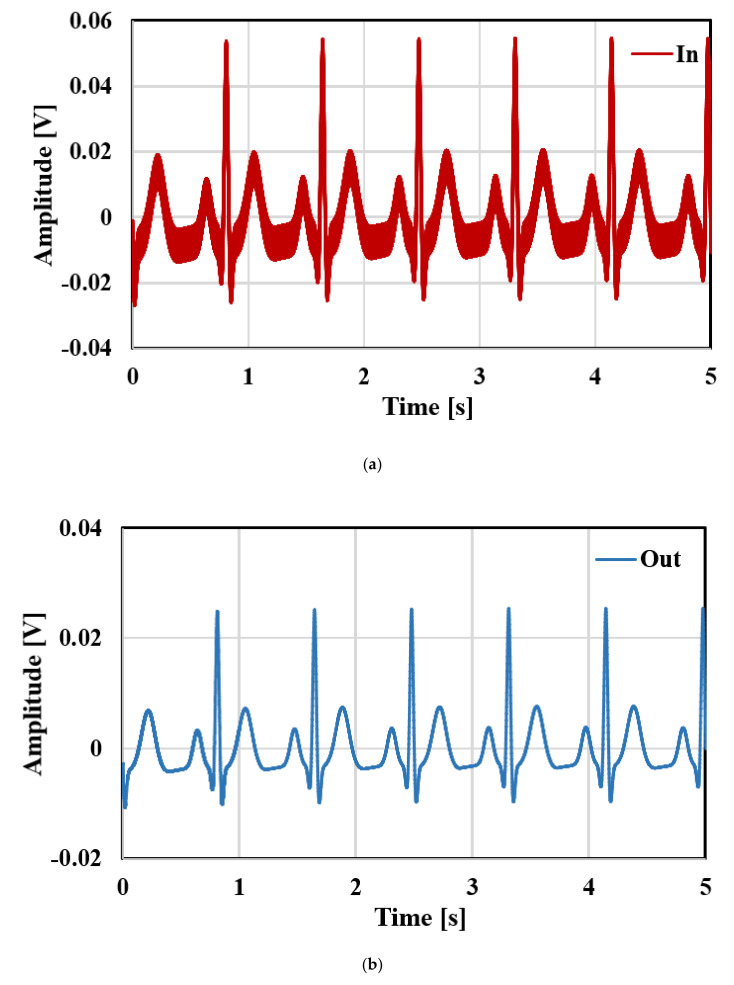
Transient response of the filter for ECG signal: (**a**) input; (**b**) output.

The Monte Carlo analysis with 200 runs was performed for the filter gain and cut-off frequency as shown in Figure 15 and Figure 16, respectively. The mean value of the gain was −6.23 dB with standard deviation of 0.14 dB, while the mean value of the cut-off frequency was 251.7 Hz with standard deviation of 4.9 Hz. Figure 17 shows the output referred noise density of the filter. The integrated in-band noise between 0.1 Hz to 250 Hz shows that the output referred noise is 77 μV_rms_. Figure 18 shows the performance of the proposed filter in processing the ECG signal where (a) depicts the ECG signal with a distortion signal (5 mV/500 Hz) that was applied at the input of the filter and (b) depicts the filtered output signal.

**Figure 19:**
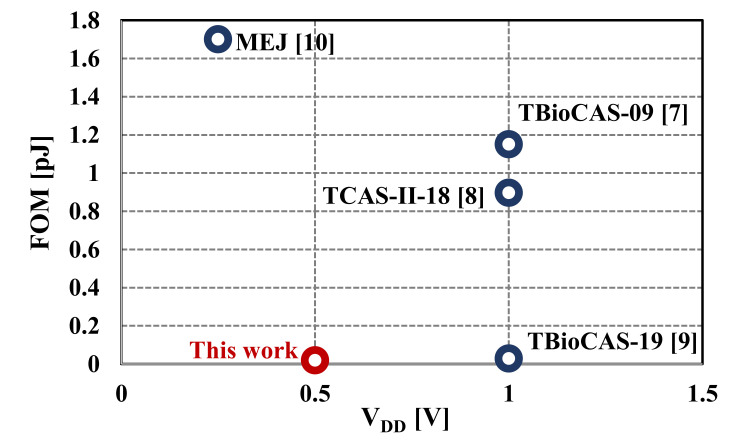
Figure-of-merit (FOM) against V_DD_ of the fifth-order low-pass filters.

The summary and comparison between the proposed filter and some previous works are shown in Table 1. Only the fifth-order Butterworth low-pass filters simulated by the LC-ladder type filter and suitable for ECG signal acquisition [7,8,9,10] have been selected for comparison. From Table 1, it is clear that the proposed filter has a lower number of active devices, power consumption, and figure-of-merit (FOM). Finally, the FOM versus V_DD_ of fifth-order low-pass filters are shown in Figure 19. Compared with the works in [7,8,10], the proposed filter offers clearly better FOM. The FOM is even slightly lower than the one in [9] with half the value of V_DD_. It is worth noting that the estimated chip area of 2-inputs and 3-inputs OTA based on the MIGD technique is increased by approximately 5% and 8%, respectively, compared to that of a single-input conventional OTA with the same transistor dimensions. This confirms the advantage of this technique of saving chip area. Note, a similar conclusion of this advantage based on experimental results is stated in [11]. The small chip area of the proposed filter is evident in Table 1 compared with that of [10] that used off-chip capacitors for filter realization.

## 4. Conclusions

In this paper, a fifth-order Butterworth low-pass filter using multiple-input OTA was proposed. The design proves that the number of OTAs for realizing the fifth-order low-pass filter architecture can be reduced using multiple-input OTAs. This entails the reduction of both the power consumption and the active area. Comparison with other designs in the literature shows that the proposed structure is the most beneficial, regarding the number of active devices and power consumption. The proposed filter was simulated with a 0.18 µm CMOS process and supplied with 0.5 V, which entailed operation in a subthreshold region. Simulation results including PVT corner and Monte Carlo (MC) analyses confirmed the robustness of the design.

## Figures and Tables

**Table 1:** Performance comparison between the proposed filter and other fifth-order low-pass filters for ECG signal acquisition.

Symbol	This Work	MEJ (2019) [10]	IEEE TBioCAS (2019) [9]	IEEE TCAS-II (2018) [8]	IEEE TBioCAS (2009) [7]
V_DD_ [V]	0.5	0.25	1	1	1
Tech [um]	0.18	0.13	0.18	0.18	0.18
V_TH_ [V]	0.5	0.44	0.5	0.5	0.5
Order (N)	5	5	5	5	5
No. of active device	5 MIGD-OTAs	6 FDDTAs	6 OTAs	6 OTAs	11 OTAs
Structure	G_m_-C fully-diff.	G_m_-C fully-diff.	G_m_-C fully-diff.	G_m_-C fully-diff.	G_m_-C fully-diff.
BW [Hz]	250	100	250	250	250
IRN [µV_rms_]	167	4.7	134	100	300
DR [dB]	63.24	57.00	61.2	49.9	50
Power (P) [nW]	34.65	603	41	350	453
FOM = P/(N * BW * DR) [pJ]	0.0191	1.7	0.0286	0.896	1.15
LV capability = V_TH_/V_DD_ * 100 [%]	100	176	50	50	50
Area [mm^2^]	0.08 (estim.) (off-chip cap.)	0.67 (off-chip cap.)	0.24	0.12	0.13
Obtained results	Simulation	Measured	Measured	Measured	Measured

**Figure A1:**
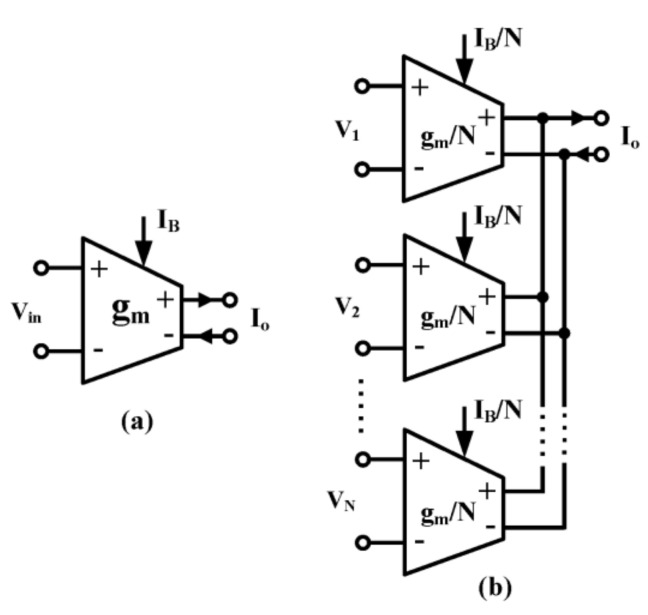
Single-input fully-differential OTA (**a**) and multiple-input fully-differential OTA (**b**).

## Appendix A

Let us compare noise properties of a fully-differential OTA in Figure A1a, biased with current *I_B_*, and a multiple-input OTA composed of *n* identical OTAs of the same structure, but biased with currents of *I_B_/n* (Figure A1b). For simplicity, let us consider only the thermal noise.

The mean-square value of the output noise current of the OTA in Figure A1a, operating in a weak-inversion region can be expressed as:

(A1) $$\bar{{𝚤}_{n}^{2}}=2qI_{B}A$$

where *q* is the electron charge and *A* is a constant depending on the particular structure of the OTA. Consequently, the input referred noise is given by:

(A2) $$\bar{v_{n}^{2}}=\frac{2qI_{B}A}{g_{m}^{2}}$$

The output noise current of each OTA in Figure A1b is:

(A3) $$\bar{{𝚤}_{n}^{2}}=2q\frac{I_{B}}{N}A$$

However, the total output noise current, equal to the sum of N output currents, is the same as that for the reference OTA in Figure A1a. If the total output noise is referred to one input, we obtain:

(A4) $$\bar{v_{n𝚤}^{2}}=\frac{\bar{{𝚤}_{n}^{2}}}{{\left(\frac{g_{m}}{N}\right)}^{2}}=\frac{2qI_{B}A}{g_{m}^{2}}N^{2}$$

Thus, the rms value of the input noise is given by:

(A5) $$\sqrt{\bar{v_{n𝚤}^{2}}}=N\sqrt{\frac{2qI_{B}A}{g_{m}^{2}}}$$

If a noiseless passive voltage divider, with N inputs, and a voltage gain of 1/N from each input is added at the input of the OTA in Figure A1a, then the rms value of the i-th input referred noise voltage is given by the same equation, namely, the i-th input-referred noise is the same as that for the OTA in Figure A1b.

If we define the dynamic range as the ratio of the maximum input rms voltage, limited by an assumed level of nonlinear distortion (*V_inmax_*) to the *i*-th input referred noise, then for the multiple-input OTA in Figure A1b we have:

(A6) $$DR_{mi}=\frac{V_{inmax}}{\sqrt{\bar{v_{n𝚤}^{2}}}}$$

Since in the subthreshold region the linear range of a differential pair does not depend on the biasing current, then for the OTA in Figure A1a, with an additional passive voltage divider, the linear range will be extended N times, and the DR will be:

(A7) $$DR_{pd}=N\cdot \frac{V_{inmax}}{\sqrt{\bar{v_{n𝚤}^{2}}}}=N\cdot DR_{mi}$$

Hence, the dynamic range of the OTA with a passive, noiseless voltage divider at the input is N times as large as that for the OTA composed of N identical OTAs, biased with N-times lower current. Similar proof could be concluded for flicker noise, however, in such a case not only the total biasing current, but also the total areas of transistor channels should be equal for the two compared circuits, i.e., the transistor channel areas of each OTA in Figure A1b should be N times smaller than for that of the OTA in Figure A1a.
